# Metabolic and genetic mechanisms of metabolic dysfunction-associated steatotic liver disease: an integrative perspective from molecular pathways to clinical challenges

**DOI:** 10.3389/fendo.2025.1639064

**Published:** 2025-09-10

**Authors:** Jingyuan Ma, Yanna Ma, Xing Wan, Junchen Li, Yunshu Zhang, Jifeng Liu, Yunhai Gao

**Affiliations:** ^1^ The First Clinical Medical College, Liaoning University of Traditional Chinese Medicine, Shenyang, China; ^2^ The First Affiliated Hospital of Dalian Medical University, Dalian, China

**Keywords:** MASLD, metabolic dysregulation, genetic polymorphisms, clinical management, precision medicine

## Abstract

Metabolic dysfunction-associated steatotic liver disease (MASLD) is now the most common chronic liver condition worldwide, closely linked to obesity, insulin resistance, and metabolic syndrome. It spans a spectrum from simple steatosis to metabolic dysfunction-associated steatohepatitis (MASH), fibrosis, and hepatocellular carcinoma. This review examines the core metabolic disruptions—particularly in lipid, glucose, bile acid, amino acid, and iron metabolism—that drive MASLD pathogenesis. It also explores how genetic variants such as PNPLA3, TM6SF2, GCKR, HSD17B13, and MBOAT7 contribute to disease susceptibility and variability in clinical outcomes. The interaction between genetic background and metabolic stress is central to the heterogeneity seen in disease progression and treatment response. We further discuss persistent clinical challenges and summarize recent advances in drugs, natural compounds, and microbiota-based strategies. Finally, we highlight the promise of multi-omics approaches to better stratify patients and personalize management. A clearer understanding of the molecular and clinical complexity of MASLD will be key to developing more effective and individualized strategies for diagnosis and treatment.

## 1 Introduction

Metabolic dysfunction-associated steatotic liver disease (MASLD) represents a spectrum of hepatic steatosis occurring in the absence of significant alcohol consumption or secondary causes of liver injury (1). Compared with the previous terminology, the current designation of MASLD is considered more appropriate, as it better emphasizes the central role of metabolic dysfunction in disease onset and progression (2, 3). A recent meta-analysis involving over 78 million participants from 38 countries estimated the global prevalence of MASLD at 30.2%, with higher rates in South America (34.0%) and Asia (30.9%) (4). Such a vast affected population underscores the urgent need for improved diagnostic and therapeutic strategies. As prevalence continues to rise globally, attention has shifted from its hepatic manifestations to its broader systemic impact. Beyond hepatic complications such as metabolic dysfunction-associated steatohepatitis (MASH), cirrhosis, and hepatocellular carcinoma (HCC), MASLD is now recognized as a multisystem disease (5). It is closely associated with a range of extrahepatic manifestations, including type 2 diabetes, cardiovascular disease, chronic kidney disease, and certain cancers (6, 7). These systemic consequences significantly contribute to the overall morbidity and mortality associated with MASLD.

In addition to lifestyle and metabolic contributors, accumulating evidence points to prenatal determinants as early-life factors influencing MASLD susceptibility (8). Maternal obesity, overnutrition, and metabolic disorders during pregnancy have been associated with increased hepatic steatosis in offspring, potentially via epigenetic programming of hepatic lipid metabolism and adipogenesis (9–11). These findings highlight a critical developmental window for future prevention and risk modification strategies.

The pathophysiology of MASLD involves complex interactions between metabolic dysregulation and genetic susceptibility. The key metabolic disturbances span lipid metabolism, glucose homeostasis, bile acid cycling, and iron handling (12–15), while common genetic variants in patatin-like phospholipase domain-containing protein 3 (PNPLA3), transmembrane 6 superfamily member 2 (TM6SF2), glucokinase regulatory protein (GCKR), hydroxysteroid 17-beta dehydrogenase 13 (HSD17B13), and membrane-bound O-acyltransferase domain-containing 7 (MBOAT7) significantly influence disease progression (16–19). This metabolic-genetic interplay creates substantial clinical heterogeneity, complicating the development of precision treatment approaches.

Persistent diagnostic and therapeutic challenges further hinder clinical management. Reliance on invasive liver biopsies persists due to limitations in non-invasive alternatives, while the absence of approved pharmacotherapeutics underscores unmet clinical needs (20). This review synthesizes current understanding of MASLD’s metabolic and genetic underpinnings, examines critical barriers to clinical management, and explores multi-omics strategies for advancing precision medicine in this complex disorder.

## 2 Metabolic dysregulation

**Figure 1:**
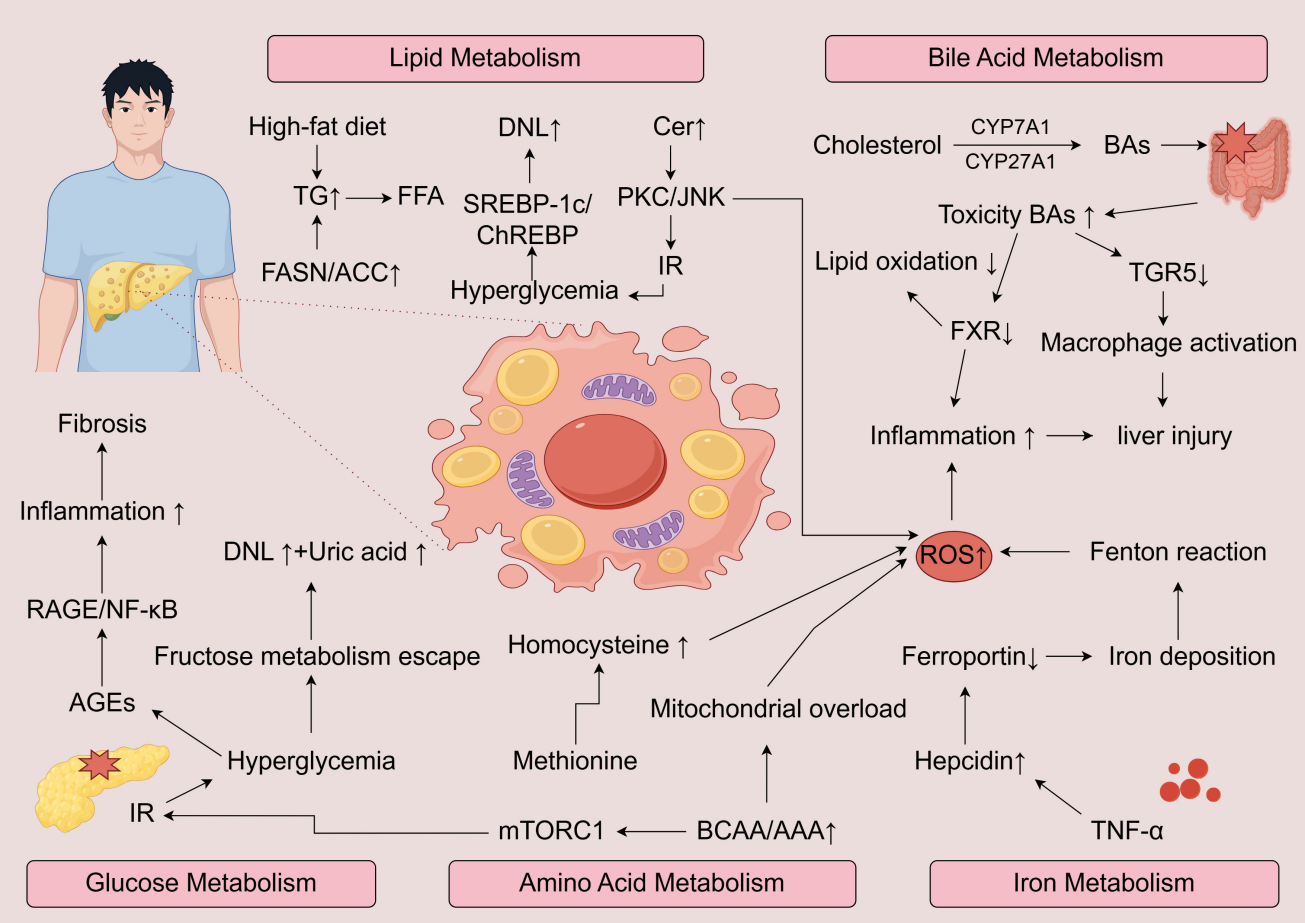
Schematic overview of the major metabolic pathways driving the progression of MASLD to MASH. Excess lipid intake enhances DNL, FFA accumulation, and ceramide synthesis, activating PKC/JNK signaling and insulin resistance. Disturbed glucose metabolism promotes AGEs-RAGE/NF-κB signaling, inflammation, and fibrosis. Altered bile acid metabolism with increased toxic bile acids and impaired FXR/TGR5 signaling exacerbates oxidative stress, macrophage activation, and hepatocellular injury. Dysregulated amino acid metabolism, including elevated BCAA/AAA and homocysteine, drives mTORC1 activation, mitochondrial overload, and ROS production. Iron overload due to hepcidin–ferroportin imbalance amplifies oxidative damage via the Fenton reaction. Together, these metabolic insults converge on excessive ROS generation and chronic inflammation, promoting hepatocyte injury, fibrosis, and disease progression.

MASLD arises from a multifaceted disruption of metabolic homeostasis involving several interrelated biochemical networks. Perturbations in lipid handling, glucose utilization, bile acid circulation, amino acid turnover, and iron metabolism collectively shape the metabolic landscape that underlies disease onset and progression. These metabolic imbalances not only contribute individually but also interact synergistically, amplifying hepatic dysfunction and fostering disease heterogeneity (**Figure 1**).

### 2.1 Aberrant lipid metabolism

Among the various metabolic abnormalities, disordered lipid metabolism is widely regarded as a central driver of hepatic steatosis. Hepatic lipid overload stems from an imbalance between fatty acid influx, endogenous lipid synthesis, mitochondrial oxidation, and lipid export via very low-density lipoprotein (VLDL) secretion (21). In MASLD pathophysiology, hepatocyte lipid accumulation originates from three sources: dietary lipids, adipose-derived free fatty acids (FFAs), and enhanced *de novo* lipogenesis (DNL) (22). In the context of insulin resistance, enhanced lipolytic activity in peripheral adipose tissue leads to elevated plasma FFA levels, which are rapidly delivered to the liver through the portal circulation (23). Simultaneously, high-fat dietary patterns further augment lipid input. Hepatic DNL becomes hyperactive under these metabolic conditions, primarily driven by transcriptional activation of lipogenic regulators such as sterol regulatory element-binding protein-1c (SREBP-1c) and carbohydrate responsive element binding protein (ChREBP). These factors stimulate the expression of enzymes including fatty acid synthase (FASN) and acetyl-CoA carboxylase (ACC), leading to increased triglyceride production (24, 25). Carbohydrate metabolism intersects with lipogenesis via glucose-activated ChREBP and UDP-glucose-mediated SREBP-1c regulation, creating a metabolic crossroad between lipid synthesis and glycogen storage (26, 27). Chronic caloric excess disrupts this balance, overwhelming hepatocellular lipid-handling capacity.

Critically, MASLD progression reflects not triglyceride accumulation per se, but rather cytotoxic lipid species buildup. Saturated fatty acids, ceramides, and free cholesterol induce organelle stress through endoplasmic reticulum dysfunction, mitochondrial damage, and lysosomal permeabilization (28). These insults converge on cell death pathways—apoptosis, necroptosis, pyroptosis—that drive steatohepatitis development (29). Among these toxic lipids, ceramides (Cer) play a pivotal role by activating protein kinase C (PKC) and c-Jun N-terminal kinase (JNK) pathways, thereby impairing insulin signaling and inducing hepatocyte apoptosis (30–32). Cer accumulation further exacerbates mitochondrial dysfunction and reactive oxygen species (ROS) production, which activate inflammatory signaling and aggravate hepatic injury (33). In addition, excessive free cholesterol accumulation in hepatocytes can provoke ER stress and mitochondrial impairment, leading to activation of the NLRP3 inflammasome, release of pro-inflammatory cytokines, and polarization of Kupffer cells toward a pro-inflammatory phenotype (34–37). These stress and immune responses, initiated by lipid metabolic dysregulation, synergistically drive the transition from simple steatosis to non-alcoholic steatohepatitis (MASH).

### 2.2 Insulin resistance and glucose metabolic dysregulation

Insulin resistance (IR) is a pivotal pathological mechanism underlying the onset and progression of MASLD. It induces systemic metabolic disturbances primarily through dysregulated interactions among adipose tissue, liver, and skeletal muscle (38). In adipose tissue, IR results in aberrant activation of hormone-sensitive lipase (HSL), leading to enhanced lipolysis and increased release of FFAs into the circulation, which are subsequently taken up by the liver (39). This process significantly contributes to hepatic lipid overload and metabolic derangement. Simultaneously, elevated levels of pro-inflammatory adipokines (e.g., TNF-α, IL-6) and decreased adiponectin further impair AMP-activated protein kinase (AMPK)-mediated fatty acid oxidation, exacerbating lipid accumulation within hepatocytes (40).

In the liver, IR presents as “selective hepatic insulin resistance”: insulin’s ability to suppress gluconeogenesis is diminished, resulting in fasting hyperglycemia, whereas its stimulatory effect on DNL remains intact or even enhanced (41). This paradox is largely mediated by persistent activation of the mTORC1/SREBP-1c axis under hyperinsulinemic conditions, which drives fatty acid synthesis and triglyceride accumulation, thereby promoting hepatic steatosis and fibrosis (42). In addition, lipid intermediates such as diacylglycerol (DAG) activate protein kinase Cϵ (PKCϵ), impairing insulin signaling and perpetuating a vicious cycle of lipotoxicity and insulin resistance (43).

In skeletal muscle, IR impairs glucose clearance due to defective GLUT4 translocation, which forces the liver to compensate by redirecting excess glucose into lipogenesis via ChREBP-dependent metabolic reprogramming—constituting a “glucose-to-lipid” maladaptive feedback loop (44, 45). Chronic hyperglycemia further exacerbates hepatocellular injury through multiple mechanisms: excessive glucose activates the polyol pathway, depleting NADPH and inducing oxidative stress, while advanced glycation end-products (AGEs) trigger inflammatory cascades via the RAGE/NF-κB axis, thereby accelerating hepatic fibrogenesis (46–48).

At the metabolic level, hyperglycemia robustly promotes DNL through the ChREBP–FASN/ACC pathway. Fructose metabolism, owing to its “metabolic bypass” nature, circumvents key regulatory steps in glycolysis and rapidly enters the tricarboxylic acid (TCA) cycle, generating excess acetyl-CoA, which fuels lipogenesis and uric acid production while inducing ER stress and oxidative damage (49–52). Additionally, acetyl-CoA derived from glucose metabolism not only serves as a substrate for DNL but also epigenetically modulates lipogenic genes such as stearoyl-CoA desaturase 1 (SCD1) and diacylglycerol acyltransferase 2 (DGAT2) via histone acetylation, thereby establishing a long-lasting “metabolic memory” that predisposes hepatocytes to persistent lipid accumulation (53, 54).

Notably, hepatocellular lipotoxic mediators such as ceramides and DAGs activate PKCϵ, leading to serine phosphorylation of insulin receptor substrate (IRS), which disrupts PI3K/Akt signaling and aggravates hepatic insulin resistance (43, 55). Concurrently, IR-associated hyperinsulinemia enhances NADPH oxidase (NOX) activity, triggering ROS generation that further suppresses insulin signaling via the JNK/IKKβ pathway (56–58). Moreover, lipid peroxidation products such as 4-hydroxynonenal (4-HNE) directly impair mitochondrial function and modify key signaling proteins, amplifying inflammatory and fibrotic responses that facilitate the transition from simple steatosis to necroinflammatory injury and hepatic fibrosis (59, 60).

### 2.3 Bile acid metabolism dysregulation

Bile acids (BAs) are bioactive molecules synthesized in the liver from cholesterol and secreted into the intestine via the bile ducts, where they facilitate the emulsification, digestion, and absorption of dietary lipids (61). BA synthesis proceeds through two primary pathways: the classical (or neutral) pathway, in which cholesterol 7α-hydroxylase (CYP7A1) serves as the rate-limiting enzyme, and the alternative (or acidic) pathway, primarily mediated by sterol 27-hydroxylase (CYP27A1) (62). Once synthesized, BAs activate the farnesoid X receptor (FXR), which induces the expression of small heterodimer partner (SHP), thereby suppressing the transcription of CYP7A1 and CYP8B1, ultimately reducing BA synthesis through negative feedback regulation (63). In addition to biosynthetic control, FXR regulates BA transport by promoting hepatic BA efflux and limiting intrahepatic BA accumulation, thus protecting against cholestasis and hepatocellular injury (64). FXR also induces intestinal fibroblast growth factor 15/19 (FGF15/19), which further acts on the liver to inhibit BA synthesis and reinforce homeostatic regulation (65, 66).

Following their secretion and intestinal metabolism, BAs undergo reabsorption and enterohepatic circulation. A portion of BAs enters systemic circulation, where they interact with nuclear and membrane-bound receptors across multiple organs. This cross-organ communication enables BAs to function not only as digestive agents but also as metabolic signaling molecules that influence lipid and glucose metabolism, energy homeostasis, and immune responses (61).

In the context of MASLD, BA metabolic dysregulation has been identified as a critical contributor to disease pathogenesis. Alterations in BA composition and impaired FXR/TGR5 signaling may disrupt hepatic lipid and glucose homeostasis, promote hepatic fat accumulation, exacerbate inflammatory responses, and accelerate liver injury (67, 68). A study shows that several bile acids are significantly elevated in patients with MASLD, suggesting their potential utility as biomarkers for distinguishing MASH from simple steatosis (69).

### 2.4 Amino acid metabolism dysregulation

An expanding body of research underscores the significant contribution of amino acid metabolic disturbances to MASLD pathogenesis (70–73). As the liver serves as a central hub for amino acid synthesis, degradation, and interconversion, perturbations in systemic amino acid profiles are frequently observed in hepatic dysfunction (74). In MASLD, elevated circulating concentrations of branched-chain amino acids (BCAAs) and aromatic amino acids (AAAs) have been consistently identified (72, 75, 76). These elevations correlate strongly with insulin resistance and altered lipid handling, thereby facilitating hepatic fat accumulation and exacerbating inflammation (77). Beyond their metabolic roles, amino acid-derived intermediates can generate reactive oxygen species and impair mitochondrial function, further disrupting insulin signaling and accelerating disease progression (78).

Among individual amino acids, glycine levels are frequently depleted in MASLD. As a precursor for glutathione biosynthesis, reduced glycine availability compromises antioxidant defenses. Supplementation with glycine has demonstrated beneficial effects on liver steatosis and inflammation in both preclinical models and clinical trials (79). Disruptions in methionine metabolism are also prominent in MASLD, often resulting in hyperhomocysteinemia and diminished glutathione production—factors that collectively enhance oxidative stress and hepatic lipid deposition (80). In pediatric MASLD populations, metabolic alterations involving methionine, tyrosine, and tryptophan pathways have been observed, suggesting that such changes may manifest early in disease evolution (81). Additionally, aberrant expression of glutamine-metabolizing enzymes has been reported in MASLD livers, potentially affecting redox regulation and modulating immune responses (82).

### 2.5 Iron metabolism dysregulation

Iron, a vital micronutrient, participates in critical physiological processes such as oxygen transport, mitochondrial respiration, and DNA replication (83). Disruption of iron equilibrium can interfere with cellular function and has been implicated in the pathophysiology of diverse conditions, including anemia, neurodegenerative diseases, and malignancies (84). Increasing evidence also supports a critical role for iron metabolism dysregulation in the development and progression of MASLD (85, 86).

Approximately one-third of MASLD patients exhibit elevated serum ferritin levels, normal or mildly increased transferrin saturation, and mild hepatic iron deposition—a constellation referred to as “dysmetabolic iron overload syndrome (DIOS) “ (87). Excessive hepatic iron accumulation promotes the generation of hydroxyl radicals via the Fenton reaction, triggering lipid peroxidation, mitochondrial dysfunction, and apoptosis (88). These events exacerbate hepatic inflammation and fibrosis, ultimately accelerating the transition from simple steatosis to MASH.

Moreover, iron overload has been closely linked to insulin resistance, potentially by impairing insulin signaling and reducing insulin sensitivity, thereby facilitating hepatic fat accumulation (89). In individuals with MASLD, pro-inflammatory cytokines—particularly tumor necrosis factor-α (TNF-α)—can induce hepatic overexpression of the iron-regulatory hormone hepcidin. Elevated hepcidin suppresses ferroportin, the principal iron exporter, thereby promoting iron retention within hepatocytes and Kupffer cells. This accumulation enhances oxidative stress and pro-inflammatory signaling, exacerbating liver injury and fibrosis (90).

### 2.6 Metabolic-genetic crosstalk

MASLD results from the interplay between systemic metabolic stressors and genetic predisposition, forming a complex disease network. Metabolic disturbances—such as insulin resistance, lipotoxicity, and bile acid dysregulation—initiate and perpetuate hepatic injury, while genetic variants influence individual susceptibility, disease progression, and therapeutic response.

For instance, the PNPLA3 I148M variant impairs lipid droplet remodeling, promotes free cholesterol accumulation, and induces mitochondrial dysfunction in hepatic stellate cells (LX-2), thereby activating them and contributing to liver fibrosis (91, 92). The TM6SF2 E167K mutation reduces VLDL secretion, heightening hepatocellular lipid stress (93). In glucose metabolism, GCKR P446L enhances DNL in hyperinsulinemic states, especially under high-glucose dietary conditions (94). MBOAT7 loss impairs phospholipid remodeling, exacerbating lipid accumulation and inflammatory signaling under hyperinsulinemic or lipotoxic conditions, thereby promoting fibrosis (95, 96). In contrast, loss-of-function variants in HSD17B13 can attenuate hepatocellular injury and inflammatory responses under metabolic stresses such as lipotoxicity and oxidative stress, thereby conferring protection against MASH and fibrosis (19, 97).

These examples highlight that genetic risk is context-dependent, with phenotypic expression shaped by the metabolic milieu. Conversely, metabolic interventions may yield variable outcomes depending on genetic background. This metabolic-genetic crosstalk explains the clinical heterogeneity of MASLD and supports a shift toward genotype-informed, metabolism-guided precision medicine. Furthermore, gene-environment interactions play a pivotal role in modulating MASLD severity. Dietary composition (e.g., high sugar or saturated fat intake), physical inactivity, and even prenatal exposures may amplify or attenuate the phenotypic effects of genetic variants such as PNPLA3 I148M or TM6SF2 E167K. Understanding these interactions is crucial for translating genetic insights into effective lifestyle or pharmacological interventions.

## 3 Genetic polymorphisms

Genetic variation critically modulates MASLD susceptibility and progression (98, 99). Key single nucleotide polymorphisms (SNPs) in PNPLA3, TM6SF2, GCKR, HSD17B13, and MBOAT7 emerge as central regulators of hepatic lipid dynamics and fibrogenesis (100). Deciphering their molecular impacts provides critical insights for personalized therapeutic development. The genetic polymorphisms of MASLD are shown in **Table 1**. These associations have been validated across diverse ethnic groups using genome-wide association studies (GWAS) and candidate gene analyses. Sample sizes vary widely across studies—from a few hundred individuals to meta-analyses involving over one million participants. Techniques such as next-generation sequencing (NGS), TaqMan genotyping, and array-based GWAS platforms have been employed to establish robust links between these polymorphisms and MASLD phenotypes.

**Table 1:** Genetic polymorphisms in MASLD: functional impacts and clinical associations.

Gene	Variant	Key Mechanism	Clinical Impact
PNPLA3	rs738409 (I148M)	• Loss of lipolytic activity → lipid droplet retention (102) • Impaired ABCG1-mediated cholesterol efflux in HSCs → mitochondrial dysfunction (104)	• 2.5× higher MASH risk (G allele) (105) • 5× increased HCC risk (GG vs. CC) (106) • Synergy with SAMM50 SNPs (109)
TM6SF2	rs58542926 (E167K)	• Reduced VLDL secretion → hepatic TG accumulation (114) • Synergy with PNPLA3/MBOAT7 variants (103)	• Increased fibrosis/HCC risk (115–117) • Paradoxical ↓ CVD risk (118)
GCKR	rs1260326 (P446L) rs780094	• Reduced GK inhibition → ↑ glucose uptake/lipogenesis (94) • Altered hepatic lipid metabolism (122–126)	• Elevated hepatic fat (121) • Interaction with PNPLA3 → MASH progression (127)
HSD17B13	rs72613567 (T>TA) rs6834314	• Loss-of-function → ↓ retinol metabolism (131) • ↑ Fibrosis risk in Han Chinese (133)	• ↓ MASLD/HCC risk (129–131) • Attenuates PNPLA3 effects (97, 136)
MBOAT7	rs641738 (C>T)	• Altered PI remodeling → TLR activation (138, 142) • ↓ ANGPTL3 → fibrosis/ASCVD link (143)	• ↑ MASH/fibrosis (141) • No pediatric association (144)

### 3.1 PNPLA3

The PNPLA3 rs738409 C>G variant (resulting in an isoleucine-to-methionine substitution at position 148, i.e., I148M) constitutes the strongest genetic risk determinant for MASLD (101). This substitution abolishes enzymatic activity, impairing lipid droplet hydrolysis and promoting intracellular retention of triglycerides and unesterified cholesterol (102). The pathogenic variant accumulates on lipid droplets, forming dysfunctional aggregates that prevent lipid release—a direct mechanistic link to hepatic steatosis (103).

Beyond steatosis, the I148M variant also impairs cholesterol handling in hepatic stellate cells (HSCs), specifically by reducing ABCG1-mediated cholesterol efflux. The resulting intracellular cholesterol buildup triggers mitochondrial dysfunction—evidenced by diminished ATP synthesis, elevated ROS, and compromised mitochondrial membrane potential—which collectively activate HSCs and promote fibrogenesis (104). Epidemiological analyses have linked the I148M variant to a significantly elevated risk of advanced liver disease. Carriers of the G allele exhibit an approximately 2.5-fold higher likelihood of developing MASH (105). Moreover, patients with the GG genotype show approximately five-fold increased HCC risk relative to CC individuals (106).

Importantly, the pathological influence of the PNPLA3 I148M variant extends beyond MASLD, suggesting broader implications across metabolic and fibrotic liver conditions. In patients with chronic hepatitis C, this polymorphism has also been associated with more severe steatosis and fibrosis, indicating its broader relevance across liver diseases (107, 108). Additionally, the combined polymorphisms in PNPLA3 and SAMM50—specifically four SNPs—have been linked to an increased risk of MASLD and elevated serum aspartate aminotransferase (AST)/alanine aminotransferase (ALT) levels, suggesting more severe hepatic injury (109). SAMM50 encodes a mitochondrial sorting and assembly machinery protein essential for maintaining mitochondrial structure and respiratory function (110). Variants in SAMM50 may disrupt mitochondrial integrity and enhance oxidative stress, thereby exacerbating hepatic steatosis and inflammation in synergy with PNPLA3 mutations. Collectively, the PNPLA3 I148M variant represents not only a major genetic driver of MASLD and its complications but also a potential biomarker for fibrosis and HCC risk, as well as a promising target for precision therapeutics (111, 112).

### 3.2 TM6SF2

TM6SF2 gene, located on chromosome 19p13.3, encodes a protein involved in hepatic lipid metabolism and has been strongly implicated in MASLD pathogenesis (113). A notable SNP, rs58542926, results in a glutamate-to-lysine substitution at position 167 (E167K) and is recognized as an independent risk factor for hepatic steatosis. This variant impairs the function of TM6SF2, reducing the efficiency of very low-density lipoprotein VLDL lipidation and secretion, which in turn causes triglyceride buildup within hepatocytes and promotes fatty liver developmen (114).

Carriers of the E167K variant not only face an elevated risk of MASLD but are also more prone to developing liver fibrosis and HCC (115–117). Additionally, emerging evidence suggests that TM6SF2 interacts synergistically with other genetic variants, including those in PNPLA3 and MBOAT7, further aggravating hepatic lipid accumulation, inflammation, and fibrotic progression (103).

Curiously, despite its association with liver disease, the E167K variant correlates with lower circulating triglyceride levels and a reduced risk of cardiovascular disease (CVD) (118). This paradox points to a potential metabolic trade-off, wherein hepatic fat retention occurs alongside decreased systemic lipid availability, possibly conferring some cardiovascular benefit. Nonetheless, the broader metabolic implications of this relationship remain incompletely understood and merit further investigation.

### 3.3 GCKR

GCKR gene encodes a critical regulator of glucose metabolism in hepatic and pancreatic tissues. By reversibly binding glucokinase (GK), GCKR modulates GK’s localization and enzymatic activity, serving as both a metabolic sensor and a safeguard against excessive glucose flux (94). Through this mechanism, GCKR contributes to the balance between glucose utilization and lipid synthesis, and its dysfunction can predispose the liver to metabolic stress and steatosis.

Genetic variants in GCKR have garnered attention due to their associations with diverse metabolic traits, including elevated fasting triglycerides, altered insulin sensitivity, and increased risk of MASLD (119–121). Among the most studied are rs1260326, which leads to a proline-to-leucine substitution at position 446 (P446L), and rs780094. Both variants have been robustly linked to hepatic fat accumulation and MASLD susceptibility (122). Functionally, the P446L variant reduces GCKR’s inhibitory effect on GK, enhancing hepatic glucose uptake and subsequent lipogenesis (94, 123). This, in turn, activates gluconeogenesis and *de novo* lipogenesis pathways, thereby promoting intracellular lipid accumulation and hepatic steatosis.

Another notable variant, rs780094, although located in an intronic region, modulates GCKR expression and has been linked to altered hepatic lipid metabolism (124–126). Carriers of this variant tend to exhibit elevated serum triglycerides and an increased risk of hepatic fat accumulation.

Interestingly, a population-based study in Turkey demonstrated that polymorphisms in both GCKR (rs1260326) and PNPLA3 (rs738409) are significantly associated with an elevated risk of MASH (127). Among them, the rs738409 variant appears to exert a stronger influence on disease progression.

### 3.4 HSD17B13

Hydroxysteroid 17-beta dehydrogenase 13 (HSD17B13) is a lipid droplet-associated hepatic enzyme with retinol dehydrogenase activity, involved in the metabolism of retinol (128). Genetic variants in HSD17B13, particularly the rs72613567 (T>TA) splice site insertion, have been extensively investigated in relation to MASLD (19). This variant results in a loss-of-function protein, which may mitigate hepatic inflammation and fibrosis by modulating retinoid metabolism, and has been associated with a reduced risk of both MASLD and HCC (129–131).

Studies have shown that both the rs72613567 and rs6834314 variants are negatively associated with MASLD and MASH, and correlate with a lower incidence of adverse hepatic outcomes in multiethnic Asian cohorts (132). However, in the Han Chinese population, the rs72613567:TA variant has been reported to be associated with an increased risk of liver fibrosis, suggesting a potential ethnic-specific effect (133). Moreover, a retrospective study found that the rs72613567:TA variant does not confer protection in advanced chronic liver disease and is associated with an increased risk of decompensation and mortality (134). Intriguingly, this variant may exert a protective effect against alcohol-induced liver damage, particularly in specific populations such as Han Chinese (135).

The interaction between HSD17B13 and other MASLD-associated genes, such as PNPLA3, has also attracted attention. Evidence suggests that the rs72613567:TA variant may attenuate the deleterious hepatic effects of the PNPLA3 I148M mutation, as reflected by lower serum transaminase levels and reduced hepatic inflammation (97). In a Japanese MASLD cohort, carriers of the HSD17B13 rs6834314 G allele exhibited a diminished impact of the PNPLA3 rs738409 GG genotype on the development of advanced fibrosis, further supporting a modifying role of HSD17B13 in genetic susceptibility to liver disease (136).

### 3.5 MBOAT7

MBOAT7 gene encodes a critical acyltransferase involved in the remodeling of phosphatidylinositol (PI), a key component of membrane phospholipids (137). MBOAT7 is highly expressed in hepatocytes and plays an essential role in maintaining membrane lipid composition and regulating intracellular signaling pathways (138). A functional variant, rs641738 C>T, has been strongly associated with MASLD susceptibility and progression in several large GWAS (139, 140).

The T allele of rs641738 is associated with decreased MBOAT7 expression, leading to reduced incorporation of arachidonic acid into PI and altering hepatic membrane lipid compositionn (96, 138). This promotes hepatic lipid accumulation and increases the risk of inflammation and fibrosis. A meta-analysis involving over one million participants found that this variant is significantly associated with increased liver fat content, elevated serum ALT levels, and a higher prevalence of MASH and advanced fibrosis—particularly among individuals of European descent (141).

In a multicenter liver biopsy cohort, the rs641738 T allele was positively correlated with fibrosis severity but showed no significant association with hepatic steatosis (104). In addition, loss of MBOAT7 function may activate the Toll-like receptor (TLR) signaling pathway and enhance the pro-inflammatory response of hepatic macrophages, further exacerbating liver injury and fibrogenesis (142). Among Han Chinese individuals, carriers of the rs641738 T allele exhibit reduced serum levels of angiopoietin-like protein 3 (ANGPTL3), which is associated with increased fibrosis severity and may mechanistically link MASLD with atherosclerotic cardiovascular disease (ASCVD) (143).

However, in contrast, another study reported no significant association between rs641738 and MASLD risk in overweight or obese children, suggesting that the genetic effects of MBOAT7 may vary by age and ethnicity (144). Collectively, current evidence supports a pivotal role for MBOAT7 in the pathogenesis of MASLD while underscoring the genetic heterogeneity of this variant across diverse populations.

## 4 Clinical challenges: from diagnosis to personalized treatment

Despite significant advances in understanding the pathophysiology of MASLD, numerous challenges persist in clinical practice (**Table 2**). From accurate diagnosis to individualized treatment, each step is hindered by technical limitations and implementation barriers, which impede early disease identification and restrict effective risk stratification and management of high-risk individuals.

**Table 2:** Clinical challenges in MASLD: from diagnosis to personalized treatment.

Category	Main Issue	Current Status and Limitations	Key References
Diagnostic Challenges	Liver Biopsy	Gold standard; invasive, sampling variability	(145, 146)
	Imaging (MRI-PDFF, FibroScan)	MRI-PDFF accurate but costly; FibroScan BMI-dependent	(147–150)
	Serologic Scores (FIB-4, NFS, ELF)	Limited accuracy near thresholds	(151–153)
	Liquid Biopsy (cfDNA, miRNAs)	Investigational, needs validation	(154)
Heterogeneity & Stratification	Genetic Polymorphisms	Variants like PNPLA3, TM6SF2, and GCKR influence susceptibility and treatment response	(98–100, 156, 157)
	Lifestyle Intervention Variability	Diet and exercise are first-line, but response varies; some patients show minimal improvement	(158, 159)
	Gut Microbiota Influence	Metabolites (SCFAs, BAs, LPS) regulate liver pathology via gut-liver axis; lack of standardized stratifiers	(160–165)
Lack of Effective Pharmacotherapy	Approved Drug	Resmetirom approved in 2024 for non-cirrhotic MASH; limited scope and long-term safety concerns	(168, 169)
	Pipeline Drugs	Aldafermin, ZSP1601, Saroglitazar, etc. under trial; some show limited or inconsistent efficacy	(170–176)
	Natural Compounds	Curcumin, quercetin, ginsenoside Rg1 show multi-target effects; low oral bioavailability and limited clinical data	(178–186)

### 4.1 Diagnostic challenges

Liver biopsy remains the gold standard for diagnosing MASLD and MASH due to its ability to directly assess steatosis, inflammation, and fibrosis (145). However, its clinical application is limited by invasiveness, risk of complications, sampling variability, and high cost (146). Consequently, non-invasive diagnostic approaches have gained traction. Imaging modalities such as MRI–proton density fat fraction (MRI-PDFF) and transient elastography (FibroScan) demonstrate good accuracy in evaluating hepatic steatosis and fibrosis (147, 148). While MRI-PDFF is constrained by limited accessibility and cost (149). FibroScan may yield less reliable results in obese individuals or those with hepatic inflammation (150). Serum-based scores including Fibrosis-4 (FIB-4) and the MASLD Fibrosis Score (NFS) are commonly used to estimate fibrosis, but their accuracy diminishes near threshold values, particularly in elderly patients or those with metabolic syndrome, leading to a risk of underdiagnosis (151–153). Emerging liquid biopsy tools, such as circulating free DNA and microRNAs, offer promise as minimally invasive biomarkers, yet remain in the exploratory stage due to limited sensitivity, specificity, and validation in large cohorts (154).

### 4.2 Challenges in addressing heterogeneity and stratification strategies

MASLD is a highly heterogeneous metabolic disorder, with a wide spectrum of histopathological phenotypes ranging from simple steatosis (SS) to MASH, cirrhosis, and HCC (155). This heterogeneity manifests not only in the rate and severity of disease progression but also in the variability of patient responses to treatment. As such, a “one-size-fits-all” therapeutic approach is insufficient to address the complexity of disease presentations in clinical practice.

Genetic background plays a pivotal role in driving this heterogeneity. As previously discussed, several genetic polymorphisms—including PNPLA3, TM6SF2, GCKR, and others—contribute to interindividual and interethnic differences in disease susceptibility and treatment response. For instance, patients carrying the PNPLA3 I148M variant exhibit greater reductions in ALT levels following semaglutide therapy (156). Similarly, rs738409 has been associated with variations in HbA1c response to dulaglutide, with potential sex-specific effects (157). These findings underscore the necessity of incorporating genetic profiling into personalized treatment strategies for MASLD.

Lifestyle interventions—including low-carbohydrate diets, Mediterranean-style diets, and structured physical activity—remain the cornerstone of MASLD management. While these approaches improve insulin sensitivity and reduce hepatic fat in many patients, responses are variable (158). Notably, some individuals fail to achieve significant reductions in hepatic steatosis despite strict adherence to lifestyle modification, suggesting that monotherapy may be insufficient in certain subgroups (159).

In recent years, the gut microbiota has emerged as a key contributor to MASLD heterogeneity. Significant interindividual differences exist in microbial composition, metabolite profiles, and microbiota-host interactions, all of which influence disease onset and progression (160). Microbial metabolites—including short-chain fatty acids (SCFAs), BAs, amino acid-derived compounds, trimethylamine-N-oxide (TMAO), and endogenous ethanol—modulate hepatic lipid metabolism, inflammation, and fibrosis via the gut-liver axis (161, 162). Disruption of the intestinal barrier can facilitate lipopolysaccharide (LPS) translocation, triggering hepatic inflammation and accelerating the transition from SS to MASH (163). Moreover, specific microbial genera such as Bacteroides and Prevotella produce SCFAs that regulate hepatic lipogenesis and energy homeostasis (164). The gut microbiota also modulates bile acid signaling through FXR and TGR5, thereby influencing hepatic inflammatory and fibrotic responses (165).

Despite growing interest, microbiota-based stratification strategies face several barriers, including the lack of highly specific microbial biomarkers, significant interethnic and geographic variation, and the complex nature of host-microbiota interactions (166, 167). These limitations currently hinder the clinical implementation of precision diagnostics and personalized interventions based on gut microbial profiling in MASLD.

### 4.3 Lack of effective pharmacotherapy

In March 2024, the U.S. Food and Drug Administration (FDA) granted accelerated approval to Resmetirom (brand name: Rezdiffra) for the treatment of adults with non-cirrhotic MASH, marking the first approved therapy for this indication and a significant milestone in the treatment of MASLD (168). However, its therapeutic scope is largely confined to patients with moderate to advanced fibrosis. Effective treatments for early-stage MASLD, decompensated cirrhosis, or patients with complex metabolic comorbidities remain lacking. Furthermore, long-term safety data are still limited, with concerns about potential cardiovascular risks and thyroid dysfunction requiring further investigation (169).

Beyond Resmetirom, several synthetic compounds are under clinical development targeting key MASLD pathways. Aldafermin (NGM282), an analog of fibroblast growth factor 19 (FGF19), has shown anti-steatotic and anti-fibrotic effects in early trials, but failed to produce significant dose-dependent improvements in fibrosis or MASH resolution in the phase IIb ALPINE 2/3 study (170–172). ZSP1601 (pan-phosphodiesterase inhibitor) reduced hepatic fat in Phase I/II, though long-term efficacy requires validation (173, 174). Other agents, such as the peroxisome proliferator-activated receptor (PPAR) agonist Saroglitazar, the apoptosis signal-regulating kinase 1 (ASK1) inhibitor Selonsertib, and the C-C chemokine receptor types 2 and 5 (CCR2/CCR5) antagonist Cenicriviroc, have shown limited or inconsistent efficacy in clinical trials, hindering broader application (175–177).

Natural compounds have attracted growing interest due to their multi-target mechanisms and lower toxicity profiles. Several phytochemicals—including curcumin, quercetin, and ginsenoside Rg1—have been shown to modulate lipid metabolism, reduce oxidative stress, and suppress inflammation through diverse molecular pathways (178–181). Curcumin inhibits the NF-κB signaling pathway, reducing pro-inflammatory cytokine expression, and has been shown in ApoE−/− mouse models to improve intestinal barrier integrity, lower endotoxin levels, and attenuate steatosis via the TLR4/NF-κB axis (182, 183). Quercetin activates the AMPK pathway, promoting fatty acid β-oxidation and reducing hepatic lipid accumulation (184). Moreover, natural products may beneficially modulate gut microbiota and restore gut-liver axis function, thereby contributing to systemic improvements in MASLD pathology (185).

Despite these advantages, clinical translation of natural products remains challenging, due primarily to poor oral bioavailability, unstable pharmacokinetics, and limited high-quality clinical data (186). Future studies should focus on enhancing delivery systems—such as nanoparticle carriers and targeted release technologies—to improve therapeutic efficacy and stability.

## 5 Future perspectives

As the complexity and heterogeneity of MASLD become increasingly apparent, precision medicine is emerging as a transformative approach to clinical management. The integration of multi-omics technologies—including genomics (e.g., PNPLA3, TM6SF2, HSD17B13), metabolomics (e.g., lipid and amino acid profiles), and microbiomics (e.g., gut–liver axis dynamics)—has enabled the construction of stratification frameworks that link molecular mechanisms to clinical phenotypes. These data-driven platforms lay the foundation for individualized risk prediction, disease monitoring, and therapeutic intervention, supporting a shift from uniform treatment paradigms to personalized care.

Building upon these insights, novel therapeutic strategies are being developed that leverage pharmacogenomic information (e.g., PNPLA3 I148M-guided therapy) and microbiome-based interventions. The latter, by reshaping bile acid signaling, immune responses, and metabolic pathways, holds promise in modulating key drivers of MASLD progression. In parallel, the therapeutic landscape is evolving toward integrated systems characterized by multi-target synergy, precision delivery technologies, and digital monitoring platforms—enabling dynamic regulation of disease trajectories and therapeutic responses.

The recent FDA approval of Resmetirom represents a therapeutic breakthrough in MASLD management, yet the disease’s multifactorial nature demands innovative strategies beyond single-target interventions. Critical areas for advancement include: (1) designing combination regimens that concurrently address lipid metabolism, inflammatory cascades, and fibrotic pathways; (2) validating non-invasive biomarkers for real-time disease monitoring; (3) developing integrative therapeutic platforms combining pharmacological agents with nutraceuticals; and (4) conducting multinational phase IV trials to establish longitudinal safety and efficacy profiles.Together, these efforts aim to bridge the gap between mechanistic discovery and clinical translation, ultimately fostering a paradigm shift toward predictive, preventive, and personalized hepatology.

## 6 Conclusion

MASLD is a multifactorial disease driven by a combination of metabolic dysfunction and genetic predisposition. Its pathogenesis involves lipid metabolic imbalance, insulin resistance, dysregulation of bile acid and amino acid metabolism, iron overload, and key genetic polymorphisms. Although advances have been made in diagnostic technologies and targeted therapeutic development, early detection and individualized treatment remain major clinical challenges. Moving forward, a precision medicine framework that integrates genomic, metabolic, and microbiome data will be essential for establishing comprehensive, stratified intervention models and achieving personalized management of MASLD—from risk prediction to mechanism-based therapy.
